# Four Decades of Cardiac Surgery in Bangladesh: A Noble Journey That Started with the Help of Japan

**DOI:** 10.31662/jmaj.2022-0148

**Published:** 2022-12-23

**Authors:** Nazmul Hosain, Farzana Amin

**Affiliations:** 1 Department of Cardiac Surgery, Chittagong Medical College & Hospital, Chattogram, Bangladesh; 2 Clinical Outcome Department, Northern Health, Prince George, British Columbia, Canada

**Keywords:** Bangladesh, cardiac surgery, pioneer, open-heart surgery, Japan, JICA

## Abstract

The first-ever open-heart operation in Bangladesh was performed on the 18th of September 1981. Although a few cases of finger fracture closed mitral commissurotomies were performed in the country in the 1960s and 1970s, full-fledged cardiac surgical services began only in Bangladesh after the establishment of the Institute of Cardiovascular Diseases at Dhaka in 1978. A Japanese team that includes cardiac surgeons, anesthetists, cardiologists, nurses, and technician came to Bangladesh and played an important role in the initiation of such a Bangladeshi endeavor. Bangladesh is a South Asian country with more than 170 million people living only in an area of 148460 square kilometers. Information was sought from the hospital records, old newspapers, books, and memoirs written by some of the pioneers. Pubmed and Internet search engines were also utilized. The principal author had personal correspondence with the available pioneering team members. The first open-heart operation was performed by visiting Japanese surgeon Dr. Komei Saji along with Bangladeshi surgeon duo Prof. M Nabi Alam Khan and Prof. S R Khan. Since then, cardiac surgery in Bangladesh has made a significant progress although it may not be enough to serve 170 million people. In 2019, twenty-nine centers performed a total of 12926 cases in Bangladesh. Remarkable advancement in cardiac surgery has been made in terms of cost, quality, and excellence in Bangladesh, but the country is lagging behind in the number of operations, affordability, and geographical distribution, which needs to be addressed for a better future.

## Introduction

On the 18th of September 1981, the first-ever open-heart operation in Bangladesh was performed. September 2022 marks the 41st anniversary of this auspicious occasion. Bangladesh is a South Asian nation with a large population. More than 170 million people are living here in 148460 square kilometers only. Following 190 years of British colonial rule and then being part of Pakistan for 24 years, Bangladesh emerged as an independent nation in 1971. High density of population, limited natural resources, unskilled human resources, and poor management all had burdened Bangladesh with economic crisis since birth. Thanks to the demographic dividend and a sustained economic growth for the last three decades, the country has recently been promoted from the status of least developed country (LDC) to lower middle-income group ^(1)^. Life expectancy has grown from 70.8 years to 74.6 years for female and from 68.4 years to 70.9 years for males in just one decade between 2009 and 2019 ^(2)^.

There is no proper documentation as to when and where the first closed-heart procedure was performed in Bangladesh. A few cases of finger fracture closed mitral commissurotomies (CMC) were performed in the Dhaka Medical College premises in the late 1960s and early 1970s ^(3)^. During a similar period, a few mitral valvotomies were performed at the then TB hospital, which is the predecessor of the National Institute for Diseases of Chest & Hospital (NIDCH). However, full-fledged cardiac surgical services began in Bangladesh only after the establishment of the Institute of Cardiovascular Diseases (ICVD) at Dhaka in 1978. There were no established Bangladeshi cardiac surgeons or cardiac anesthetists when the first open-heart operation was performed. A Japanese team that includes cardiologists, cardiac surgeons, anesthetists, nurses, and technicians supported by Japan International Cooperation Agency (JICA) was deployed in Bangladesh for capacity building of the local human resources ^(3)^. The objective of this study is to draw a pen-picture of those early days in order to extract the memory of the pioneering members. In addition, a brief description of the present situation and future prospect of cardiac surgery in Bangladesh will be included.

## Methodology

It is not easy to figure out the exact details of such an extraordinary event almost four decades ago. The memory of the persons concerned has been the primary source of information. Most of the pioneers are still alive. Many of them still retain sharp memory to describe the events of the first open-heart operation. The eight persons were interviewed to obtain information related to that operation. They were either a part of the pioneering team or working at ICVD during that period. Formal appointment was made, and the principal investigator interviewed the persons. At first, the person was asked to describe the early days and then the interviewer asked specific questions to bridge the gaps. The average interview time was 53 minutes. Dr. Komei Saji was interviewed via email with the help of his son. Dr. Minhaz Uddin was contacted via phone calls and emails.

The ICVD records and relevant documents have been thoroughly searched. The Operation Theater and ICU record books have been scrutinized. The books and memoirs written by some of the above have also been good sources of information. The newspapers at that time were searched for relevant news from the documentation center of the National Archive and National Library of Bangladesh. Pubmed and other Internet sources were explored in search of information related to the operation. The corresponding author had a telephone discussion with the first patient himself sometime in 2012 or 2013. However, contact with him was lost, and the latest whereabouts of the patient could not be reestablished at the time of writing of this paper.

For the description of the current and future situation of cardiac surgery in Bangladesh, information was gathered from all the centers that perform cardiac surgery. There is no national database of the cases performed here. However, Bangladesh Association of Cardiovascular and Thoracic Anesthesiologists (BACTA) compiles the data on annual performances of different cardiac surgical centers of the country and publish in their journal. This yearly publication provides an accurate picture of the numerical values of the cases performed in Bangladesh. Other contemporary publications were searched for the present status and future perspectives of cardiac surgery in Bangladesh.

## Historic Perspective

History of Western medicine in the South Asian subcontinent may be traced back to the sixteenth century, when medical personnel arrived from India by the British East India Company’s early fleets as ships’ surgeons ^(4)^. Following the Battle of Plassey in 1757, East India Company established its control over Bengal. A medical department was established in Bengal as far back as 1764, mainly for rendering medical services to the troops and officers of the East India Company. Madras General Hospital was formed at Madras (present-day Chennai) in 1679, the first western medical institute of this region. Presidency General Hospital was established in Calcutta (Kolkata) in 1796. Established in 1835, Calcutta Medical College is the first medical college of this region and of Asia as well. The corresponding hospital named Calcutta Medical College Hospital was established in 1852. The first Indian war of independence (or Sepoi Mutiny) of 1857 led to the transfer of administrative power of India from The East India Company to the British crown, and different departments of civil service were established. In 1868, a separate civil medical department was established in Bengal. In 1939, All India Institute of Hygiene and Public Health was established in Calcutta, and the first rural health training center was founded at the nearby Singur in West Bengal ^(5)^.

Established in 1946, Dhaka Medical College was the first medical college within the present-day Bangladesh territory. At the end of British colonial rule in 1947, the relatively backward eastern part of Bengal joined Pakistan as East Pakistan, which became Bangladesh after the war of independence in 1971. Following independence, the inherited health infrastructure from the British colonial and Pakistani era has undergone a major expansion ^(4)^. Cardiology as a discipline was first introduced at the then Institute of Post Graduate Medicine and Research (IPGMR) in the 1970s. However, the cardiology service rendered was very primitive. Some enthusiastic physicians tried to provide service without proper equipment and expertise, and some even more enthusiastic and brave surgeons also attempted a few cases of cardiac surgery.

While serving as the Professor of Surgery of the then IPGMR in the late 60s or early 70s, a renowned general surgeon Prof. Ali Ashraf had performed a few cases of finger fracture mitral valvotomy. IPGMR was still located at the Dhaka Medical College Hospital premises. So, these operations were performed at the DMCH Operation Theater complex although by a surgeon belonging to IPGMR. In a similar period, Dr. Mominul Haque, a general surgeon, performed a few cases of valvotomy at the then TB Hospital at Mahakhali, which is the present-day NIDCH. However, the establishment of ICVD Dhaka in 1978 introduced full-fledged cardiac surgical services in Bangladesh.

Indoor service of ICVD began in August 1978. However, it only included cardiology initially. Prof. M Nabi Alam Khan FCPS, FRCS, was the first surgeon to join ICVD. Prof. Sirajur Rahman Khan FRCS popularly known as S R Khan had driven back home from the United Kingdom and joined the Chest Hospital. These two pioneering surgeons well-known for their professional jealousy and personal friendship kept both the centers running ^(3)^. These two surgeons started working with the Japanese surgeons in preparation for the first cardiac operation in Bangladesh.

ICVD Operation Theater Number-1 was commissioned on the 14th of June 1980. On the very first day, three general operations were performed by Prof. M Nabi Alam Khan and assisted by Dr. Minhaz ^(6)^. The first operation was an excision of a fibroadenoma of the left breast of a 24-year-old patient. The anesthesia was administered by Prof. Khalilur Rahman and Dr. Nurul Islam. The first few cases of ICVD OT were not cardiac, rather included a wide variety of general surgery cases. The first true cardiac case was a closed mitral valvotomy performed by Prof. M Nabi Alam Khan on 9th July 1980. Here again, the anesthetist was Prof. Khalilur Rahman and assisted by Dr. Zaheda. Although it is impossible to point out the exact first cardiac operation, this closed mitral valvotomy may be described as the first definite and documented cardiac surgical procedure in Bangladesh ^(6)^.

Technical cooperation with Japan began in 1980. Japanese cardiologists, cardiac surgeons, anesthetists, and nurses began arriving at Dhaka. Japanese surgeons Dr. Komei Saji and Dr. Tomino started taking part in closed-heart operations along with their Bangladeshi counterparts. Some Bangladeshi surgeons, anesthetists, nurses, and technicians received training in Japan. By the month of September, the combined team was ready for the first open-heart surgery. An 18-year-old college student hailing from Sitakunda, Chittagong, diagnosed with Secundum-Type Atrial Septal Defect was selected as a suitable candidate for the first operation.

On the 18th of September 1981, the first-ever open-heart operation in Bangladesh was performed at Operation Theater Number-1 of ICVD ^(2)^. The surgical team (Table 1) comprised Dr. Komei Saji of Japan, Prof. M Nabi Alam Khan, Prof. S R Khan, and Dr. Minhaz Uddin ^(6)^. Another Japanese surgeon, Dr. Tomino and Dr. Fazlur Rahman, the then resident surgeon of ICVD, performed perfusion. The anesthetists were Prof. Khalilur Rahman and Dr. Nurul Islam ^(6)^. Other anesthetists supporting in Operation Theater and ICU included Dr. Abdul Hadi, Dr. Delowar Hossain, Dr. A Y F Ellahi Chowdhury, and Dr. Monir Hossain. Two future directors of NICVD, then Assistant Professor of Cardiology Dr. M Nazrul Islam and Assistant Registrar of Cardiology Dr. Mohibullah along with Dr. Nasiruddin Ahmed, a future Head of the Department of Cardiac Surgery working in the Department of Cardiology at that time, were present on that day and witnessed this glorious event.

**Table 1.** The Pioneering Surgical Team.

Name	Position in 1978-81	Current whereabouts (2021)
Prof. M Nabi Alam Khan	Associate Professor Cardiac Surgery, ICVD (Bangladesh)	Deceased on September 27, 2007
Prof. S R Khan	Associate Professor Cardiothoracic Surgery, IDCH (Bangladesh)	Retired from professional activities and living in Kushtia
Dr. Komei Saji	Consultant Cardiac Surgeon (Japan)	Retired from Surgery and living in Sendai, Japan
Dr. Tomino	Consultant Cardiac Surgeon and Perfusionist (Japan)	Returned to Japan
Dr. Khalilur Rahman	Associate Consultant Anesthetist and Associate Professor, ICVD (Bangladesh)	Retired from professional activities and living in Dhaka
Dr. Nurul Islam	Anesthetist, ICVD (Bangladesh)	Deceased
Dr. Minhaz Uddin	Junior Surgeon (Bangladesh)	Living in Ohio, USA
Ms. Anima Boiragi	Scrub Nurse (Bangladesh)	Retired from NICVD but engaged in a private job

At the operation table, the atrial septal defect was found a small one. It was managed by direct closure. Cross clamp time was 27 minutes. Rest of the operation went smoothly, and the patient was transferred to the ICU by 2.30 PM. The ICVD ICU was equipped with Nihon Kohden invasive cardiac monitors and IKA R120 ventilator at that time. One of the team members recalled that soon after the patient was shifted to the ICU, it was detected that half a bag of mismatched blood had been transfused to the patient ^(7)^. With mannitol, steroids, and other medications available in those days, the mismatch transfusion was managed successfully.

The Japanese surgeon Dr. Komei Saji remembers that excessive bleeding from the chest drainage tube led to reopening of the chest that night. The bleeding was noted from the aortic cannulation site, which was controlled ^(7)^. The remainder of ICU stay of the patient was smooth and uneventful. An inquiry committee formed to investigate the incident found no malafide intention or sabotage and concluded that human error was responsible for the mistake.

**Figure 1.**
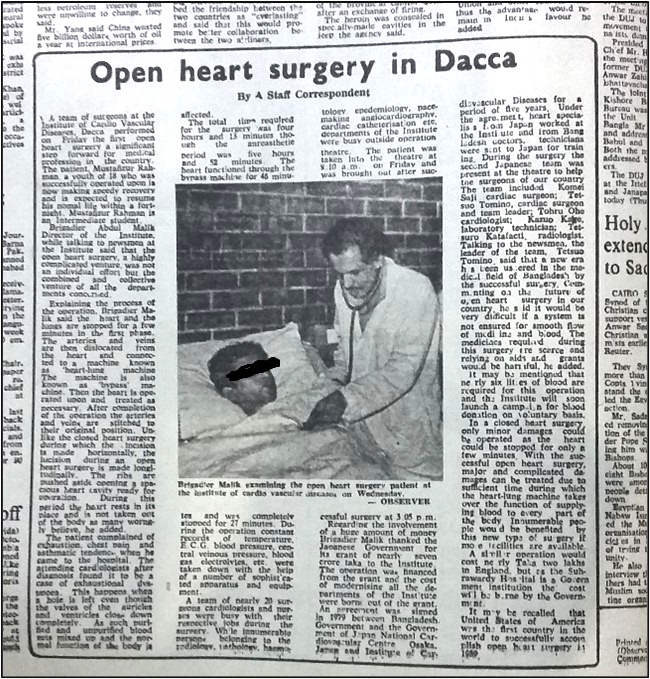
Report published in a local English daily “Daily Observer” quoted with permission obtained from its editor.

The world’s era of open-heart surgery began with Dr. John Heysham Gibbon ^(8)^, who closed the atrial septal defect of an 18-year-old girl using his invented heart lung machine on the 6^th^ of May 1953. The first open-heart surgery of the South Asian subcontinent was performed by Dr. K N Dastur at Bombay on 16^th^ February 1961 ^(9)^. Twenty years later, the first open-heart operation in 1981 was considered a real heroic act in Bangladesh. The local media flashed the news of this great achievement. A news report from a local newspaper Daily Observer has been mentioned here as a reference (Figure 1) ^(10)^. The people of Bangladesh welcomed this news with euphoria. To celebrate the thirtieth anniversary of the event, the postal department of Bangladesh published a commemorative stamp and a first day cover in 2011 on the 30th anniversary of the operation ^(11)^.

## Bangladesh Aftermath

Since 1981, cardiac surgery in Bangladesh has made a significant progress although it may not be enough to meet the demand of its 170 million population. Both the number of cardiac operations and the number of centers offering cardiac surgery have increased over the years. From just one open-heart operation in 1981, the number of cases crossed two hundred in 1997. There was a sharp increase in the number of cases after 2001. The total number of operations performed in 2016 was 11121 ^(12)^, which increased to 12926 in 2019 ^(13)^, the last year before the COVID-19 pandemic. Of these, CABGs were 67.1%, whereas valvular and congenital cases were 11.7% and 19.9%, respectively. The number of centers offering cardiac surgery was 25 in 2017 ^(12)^, which increased to 32 as of 2022. Four of these centers are run by the Government, one by the Bangladesh Army and one center is run by the autonomous medical university. The remaining 25 are private enterprises. Bangladeshi cardiac surgery centers perform almost all kinds of cardiac operations except cardiac transplantation and some extremely complicated neonatal surgeries.

## Future of Cardiac Surgery in Bangladesh

**Figure 2.**
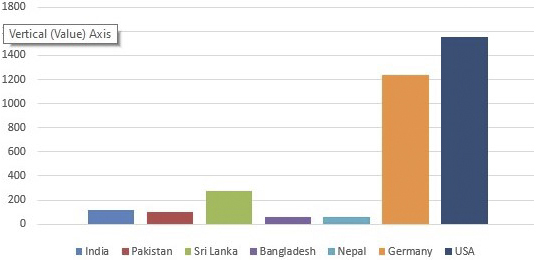
Graph showing the number of cardiac operations performed per million population in the countries of South Asia, Germany, and the USA.

Eleven thousand cardiac surgical cases per year might sound an impressive figure, but if it is performed for 170 million people, it still appears small. Figure 2 shows the estimated number of cardiac surgery cases per million population performed by the South Asian nations in 2017. India alone performs around 75% of all cardiac operations of South Asia because of its huge population size, followed by Pakistan, Bangladesh, Sri Lanka, and Nepal in the absolute numerical order. When the number of cases per million population is considered, Sri Lanka is the best performer in the region vastly out numbering its neighbors. However, compared with the western world, even the top performance of Sri Lanka appears dwarf (Figure 2).

A multi-author study covering 16 different countries was conducted to assess the required number of cardiac operations ^(14)^. The study estimated a requirement spectrum ranging from 200 operations per million in low-income countries to >1,000 operations per million in high-income countries representing the end of the epidemiological transition. The actually observed levels of cardiac surgery ranged from 0.5 per million in the assessed low- and lower middle-income countries (average 107 ± 113 per million; representing a population of 1.6 billion) to 500 in the upper-middle-income countries (average 270 ± 163 per million representing a population of 1.9 billion) ^(14)^.

Considering the findings of this study, it may be stated that although the number of operations in Bangladesh is better than that of the number in some of the low-income countries under that study, it is far less than the desired level of 200 operations per million population.

Another consideration is the geographical distribution of Bangladeshi centers. Table 2 presents the names of 32 centers currently (as of 2022) performing cardiac surgery in Bangladesh. As of 2019, the National Heart Foundation and Research Institute Dhaka has performed 2898 cases, the highest number constituting 22.8% of all the cases alone. The next two centers in terms of the number of cardiac surgeries are NICVD with 1429 (11%) and United Hospital with 1312 (10.2%) cases. Khawza Yunus Ali Medical College at Sirajganj is the first center outside Dhaka to start cardiac surgery in 2004. Of these 32 centers, 24 are located in Dhaka, the capital city. These centers together perform more than 95% of the cases, although this city proper hosts only 5% of the country’s population. This has created an imbalance in the availability of cardiac operations in already existing situation of scarcity. Bangladesh is a small country, and communication is not a big problem. However, just like the distribution of the cardiac centers, distribution of wealth has also a similar geographical distribution pattern. In 2021, the per capita GDP (nominal) of Bangladesh is US$ 2122, whereas the per capita GDP of Dhaka city is US$ 7712. The estimated per capita GDP of the rest of the country other than Dhaka city is merely US$ 1368. So, ironically, the people from the poor areas of the country must take expensive journeys far from their homes for cardiac operations. The expenditure of cardiac operations in Bangladesh is among the lowest in the world ^(15)^. The cost of CABG or a congenital surgery may range from US$ 350 in a public hospital to US$ 5000 in posh private hospitals. Even this tiny amount of US $ 350 may be very difficult to spend for most of the population. The estimated ratio of the cost of CABG and per capita GDP is 1.35. With absence of any health insurance scheme, health cost in Bangladesh is mostly out of pocket expenditure. Any cardiac surgery for most of the population turns out to be a catastrophic health event and may result in the poverty of the family ^(15)^.

**Table 2.** List of Bangladeshi Centers Offering Cardiac Surgery as of 2022.

Dhaka Division	1. National Institute of Cardiovascular Diseases, Dhaka
	2. Combined Military Hospital, Dhaka
	3. National Heart Foundation & Research Institute, Dhaka
	4. Bangabandhu Sheikh Mujib Medical University Dhaka
	5. United Hospital, Dhaka
	6. Lab Aid Cardiac Hospital Dhaka
	7. Ibrahim Cardiac Hospital & Research Institute, Dhaka
	8. Evercare Hospital, Dhaka
	9. Z H Sikder Medical College & Hospital, Dhaka
	10. Dhaka Shishu Hospital, Dhaka
	11. Metropolitan Medical Center, Dhaka
	12. Asgar Ali Hospital, Dhaka
	13. Square Hospital, Dhaka
	14. Al Helal Hospital, Dhaka
	15. Euro-Bangla Heart Hospital, Dhaka
	16. Ibne Sina Medical College & Hospital, Dhaka
	17. Sirajul Islam Medical College & Hospital, Dhaka
	18. Ayesha Memorial Hospital, Dhaka
	19. Lubana General Hospital, Dhaka
	20. Bangladesh Specialized Hospital, Dhaka
	21. Green Life Hospital, Dhaka
	22. Dhaka Medical College Hospital, Dhaka
	23. Sir Salimullah Medical College and Mitford Hospital, Dhaka
	24. Impulse Hospital, Dhaka
Chattogram Division	1. Chittagong Medical College & Hospital, Chattogram
	2. Chattogram Metropolitan Hospital, Chattogram
	3. Imperial Hospital, Chattogram
	4. Evercare Hospital, Chattogram.
Rajshahi Division	1. Khawza Yunus Ali Medical College, Sirajganj
Khulna Division	1. Khulna City Medical College, Khulna.
Rangpur Division	1. Zia Heart Foundation, Dinajpur
Sylhet Division	1. National Heart Foundation Hospital, Sylhet

As of July 2022, no center in Barishal, Mymensingh, Meghna, or Padma divisions is offering cardiac surgery.

The legacy of the cardiac surgery in Bangladesh is interesting. In 1981, Bangladesh hosted the 7th largest population of the world. These 81.7 million people were the biggest population of the world without any cardiac surgical services. With the introduction of cardiac surgery at ICVD Dhaka in 1981, the Bangladeshi people were relieved of this unglorified title indicating the lack of surgical cardiac care. However, due to poor geographical distribution, these facilities did not reach many Bangladeshis. Until 2011, there were no cardiac surgical facilities in Chittagong (Chattogram), the second biggest city in the country. Chittagong with more than 4 million population in 2011 was the biggest city of the world without any cardiac surgical service. With the beginning of cardiac surgery in a private hospital of Chittagong on 28^th^ January 2011 and in Chittagong Medical College Hospital on 10^th^ April 2012, Chittagong got rid of this undignified status. Chittagong Medical College Hospital hence became the first public medical college hospital to offer cardiac surgery anywhere in Bangladesh (Table 3).

**Table 3.** Development of Cardiac Surgery in Bangladesh.

Timeline	Event
May 5, 1953	World’s first open-heart surgery by John Gibbon in the USA
1960s and 1970s	A few closed-heart operations at IPGMR and TB Hospital
1978	Establishment of Institute of Cardiovascular Diseases
June 14, 1980	Installation of ICVD OT and first noncardiac operation
July 9, 1980	First closed-heart operation (CMC) at ICVD
September 18, 1981	First open-heart surgery of Bangladesh
2004	First cardiac surgery outside Dhaka at Khwaja Yunus Ali Hospital, Sirajganj
April 10, 2012	First open-heart operation at a public medical college hospital: Chittagong Medical College Hospital, Chattogram

Even in 2021, only four (Dhaka, Chattogram, Khulna, and Sylhet) of the eleven-city corporation areas of Bangladesh have facilities for cardiac surgery. The other seven, namely, Rajshahi (with a population of 763,952), Mymensingh (389,918), Barisal (339,308), Rangpur (307,053), Cumilla (296,010), Narayanganj (286,330), and Gazipur (213,061) currently do not have any cardiac surgical facilities. None has even the basic infrastructure to provide cardiac surgical services for their populations ^(16)^. Bangladesh has made remarkable progress in various areas over the last three decades, including primary health care. Cardiac surgery is an area where progress has been made in terms of quality and excellence, but there is still a long way to go in terms of number and geographical distribution of these operations.

## Final Words

Cardiac surgery is one of the most challenging and sophisticated areas of medical science. Once thought impossible for various obstacles, it became a day-to-day affair after the invention of the heart lung machine by John Gibbon in 1953. The first open-heart surgery of Bangladesh was performed on the 18^th^ September 1981 by a Bangladeshi-Japanese joint team. Japanese surgeon Dr. Komei Saji along with Bangladeshi surgeon duo Prof. M Nabi Alam Khan and Prof. S R Khan were the surgeons of that pioneering operation. Since then, a remarkable advancement has been made in terms of cost, quality, and excellence of cardiac surgery in Bangladesh, but the country is still lagging in number, affordability, and geographical distribution. As of early 2022, a total of 29 centers are performing more than twelve thousand cases a year, but many more are needed in order to meet the demand of 170 million people of the country.

## Article Information

### Conflicts of Interest

None

### Acknowledgement

The initial idea of recording the history of Bangladesh came while having a chat with three cardiologists. They were two former NICVD Directors Prof. A K M Mohibullah and Prof. Abdullah Al Shafi Majumder, and Dr. A K M Monwarul Islam. I am grateful to them. I pay tribute to former ICVD Professor of Anesthesia Dr. Khalilur Rahman and three more former NICVD Directors, namely, National Professor Brigadier (Rtd.) Abdul Malik, Prof. M Nazrul Islam, and Prof. A Y F Elahi Chowdhury as most of the information of this article came from them. However, some aspects of the early ICVD days I came to know while working there as an Assistant Registrar during 1996-97. I had the opportunity to listen to Prof. S R Khan, Late Prof. M Nabi Alam Khan, Prof. M Alimuzzaman, Prof. Nasiruddin Ahmed, Prof. N A Kamrul Ahsan, Prof. Asit Baran Adhikary, Prof. Faruque Ahmed, Dr. Jahangir Kabir, Late Prof. M Aftabuddin, Prof. Abul Kashem, Dr. SAM Abdus Sabur, Dr. A A Solaiman, Dr. Lutfor Rahman, and Prof. M Sharifuzzaman along with anesthetists Prof. A T M Khalilur Rahman, Late Dr. Ahsan Habib, Perfusionist Dr. Bilkis Banu, and many others. I thank them all. A 1981 news clip from Daily Observer has been used in the article. We express our gratitude to those dailies along with Mr. Syed Badrul Ahsan, the Associate Editor of Daily Observer.

After a long and painstaking search, I managed to establish contact with one of the Japanese surgeon members of the pioneering team Dr. Komei Saji. I am grateful to him for helping me with information and photographs for the article.

Finally, I offer special thanks to all the pioneers of the early days for their invaluable contribution in the development of cardiac surgery in Bangladesh.

### Author Contributions

Dr. Nazmul Hosain contributed to literature search, study design, data collection, data interpretation, writing, and editing.

Dr. Farzana Amin contributed to literature search, data interpretation, and editing.

The research was conducted at the Department of Cardiac Surgery, Chittagong Medical College & Hospital, Chattogram, Bangladesh.
